# The Role of Antimicrobial Peptides in Influenza Virus Infection and Their Potential as Antiviral and Immunomodulatory Therapy

**DOI:** 10.3390/ph9030053

**Published:** 2016-09-06

**Authors:** I-Ni Hsieh, Kevan L. Hartshorn

**Affiliations:** Department of Medicine, Boston University School of Medicine, Boston, MA 02118, USA; inhsieh@bu.edu

**Keywords:** cathelicidin, defensin, LL-37, histone, amyloid

## Abstract

Influenza A virus (IAV) remains a major threat that can cause severe morbidity and mortality due to rapid genomic variation. Resistance of IAVs to current anti-IAV drugs has been emerging, and antimicrobial peptides (AMPs) have been considered to be potential candidates for novel treatment against IAV infection. AMPs are endogenous proteins playing important roles in host defense through direct antimicrobial and antiviral activities and through immunomodulatory effects. In this review, we will discuss the anti-IAV and immunomodulatory effects of classical AMPs (defensins and cathelicidins), and proteins more recently discovered to have AMP-like activity (histones and Alzheimer’s associated β-amyloid). We will discuss the interactions between AMPs and other host defense proteins. Major emphasis will be placed on novel synthetic AMPs derived from modification of natural proteins, and on potential methods of increasing expression of endogenous AMPs, since these approaches may lead to novel antiviral therapeutics.

## 1. Introduction

IAV presents an ongoing major threat to human health and there is much yet to be learned about the role of innate immunity during IAV infection [1]. Although IAV elicits strong adaptive immune responses, it is prone to rapid genomic variation either through small incremental mutations or major changes resulting from exchange of genome segments with those of animal strains (reassortment). These genomic changes allow IAV to escape immune responses generated against prior strains. Generally, the small incremental changes lead to seasonal epidemics, whereas reassortment leads to pandemics. The presence of animal reservoirs allows introduction of avian or pig strains (or genes from these strains) into humans resulting in pandemics, as in 2009 [2]. Seasonal epidemics of influenza virus still contribute tremendous morbidity and mortality including annual mortality in the USA of ~40,000 [3]. Certain groups of individuals are more susceptible to severe outcomes of seasonal IAV: those at extremes of age, smokers, individuals with COPD, cystic fibrosis or asthma, diabetes mellitus, cardiovascular disease, or immune compromise. Some otherwise healthy young people die during seasonal epidemics, sometimes due to bacterial super-infection (e.g., note recent association of IAV with MRSA pneumonia) [4]. Pandemics cause more indiscriminate mortality in young healthy adults than seasonal IAV [5]. There is a period of 5–7 days prior to arrival of CD8+ T cells in the lung after exposure to a new IAV strain and innate defense is critical at this time.

There is clearly a need for more therapies for IAV infection. Currently there are only two classes of antiviral drugs active against IAV: inhibitors of the viral proton channel (M protein) and neuraminidase inhibitors. High level of resistance to amantadines and emerging resistance to neuraminidase inhibitors have been reported. In this review, we evaluate the potential of antimicrobial peptides (AMPs) as therapies for IAV through summarizing in vitro and in vivo antiviral and immunomodulatory activity of natural and modified forms these peptides.

## 2. Antiviral Activity of Various AMPs in Vitro and in Vivo vs. IAV

IAV is a respiratory tract infection that rarely causes viremia or direct infection of organs outside the lung. Despite this it can induce severe systemic illness largely through the production of pro-inflammatory cytokines. Mortality is most often linked to respiratory failure due to acute lung injury and/or bacterial super-infection. In addition, some deaths occur due to cardiovascular events likely triggered by the profound inflammatory state resulting from IAV infection in some vulnerable subjects. There has been extensive interest in development of antivirals for IAV, but also in designing therapies to dampen inflammatory injury induced by the virus. AMPs are attractive as potential therapies for IAV since they have antiviral and antibacterial activity and also exert immunomodulatory effects.

There are two major classes of amphipathic AMPs present in human respiratory lining fluids: defensins and cathelicidins. There is evidence that both of these classes of AMPs play a role during IAV infection. We will review the antiviral and immune modulatory activities of defensins, cathelicidins, and also other peptides that have other important functions but also act as AMPs (e.g., histones and Alzheimer’s associated amyloid beta). We will then discuss novel modified versions of AMPs synthesized with the aim of increasing antiviral activity. Finally, we will review potential means of inducing increased production of endogenous AMPs as an approach to antiviral treatment.

### 2.1. Defensins and Influenza

There are two major classes of defensins: α- and β-defensins. One group of α-defensins are packaged in neutrophil granules and these are termed human neutrophil peptides (HNPs) 1–4. The HNPs are very likely to interact with IAV in vivo since neutrophils predominate in the early infiltrate in the IAV infected airway and play a pivotal role in initiation of the immune response to the virus. HNPs are also displayed on neutrophil extracellular traps (NETs), which are formed in response to IAV infection in vitro [6] and in vivo [7]. Another group of α-defensins is expressed by epithelial cells, predominantly in the gut and genitourinary tracts. These are termed human defensins (HDs) 5 and 6. There is less reason to believe that HDs play a role in vivo during IAV infection, although they are of interest for their strong antiviral activity [8]. β-defensins are expressed predominantly by epithelial cells and are relevant in particular to IAV since they are expressed by the respiratory epithelium.

#### 2.1.1. HNPs and IAV

These have strong neutralizing activity for many IAV strains [8,9,10]. The mechanisms of antiviral activity of HNPs have not been fully elucidated. We have found that HNPs induce viral aggregation and inhibit infectivity mainly through direct interactions with the virus [8,10,11,12]. In our studies, incubating epithelial cells with defensins pre- or post-infection had minimal inhibitory activity and direct incubation of HNPs with the virus was needed. In contrast, Salvatore et al. have found that HNPs also inhibit IAV through binding to epithelial cells and inhibition of protein kinase C [9]. In addition, the viral binding activity of defensins is potentiated by formation of multimolecular assemblies of defensins [8,13,14]. This may help to account for viral aggregating activity of defensins. Unlike the collectins and other proteins that bind the viral hemagglutinin (HA), HNPs do not inhibit HA activity of IAV [10]. Mice do not have neutrophil α-defensins but have other antimicrobial peptides that may play a similar role; hence, it has not been possible to use mouse models to test the role of neutrophil α-defensins.

#### 2.1.2. β-Defensins and IAV

Human β-defensins (HBDs), are produced by respiratory epithelial cells either constitutively or in response to inflammatory stimuli and also inhibit IAV [8,15,16]. The HBDs are less potent as direct inhibitors of IAV than the HNPs; however, they may have important immunomodulatory roles during IAV infection as well. Ryan et al. have demonstrated that mice lacking mouse β defensin 1 (mBD1) have more severe lung inflammation when infected with IAV although viral titers were not different compared to control mice [16]. This paper also demonstrated that IAV infection increases production of mBD1 by plasmacytoid dendritic cells. Further studies of the in vivo contributions of this and other β-defensins during IAV infection will be of great interest.

#### 2.1.3. Retrocyclins

Of interest, there is a third class of defensins called θ-defensins or retrocylins (because of their cyclic nature) that are expressed in primates but not humans and have very strong anti-IAV activity [8,17]. The retrocyclins, like HNPs, can induce aggregation of IAV and they appear to have stronger intrinsic antiviral activity than HNPs [8]. As with HNPs, it was necessary to directly incubate the virus with the retrocyclins to achieve optimal inhibition of infectivity in our studies. There is evidence that retrocyclins and other defensins can act as lectins binding to viral carbohydrates [18,19], and this may be an important contributor to their activity against viruses.

#### 2.1.4. Potential for Paradoxical Activity of Defensins

HD5 and HD6 have been found to promote infection by human immunodeficiency virus (HIV) in vitro by increasing viral entry [20]. These defensins also counteract anti-HIV effects of polyanion microbicides [21]. HNP1 was also reported to increase HIV infectivity by facilitating transfer of the virus across epithelial barriers [22]. Note that other studies show an ability of these defensins to inhibit HIV [23,24,25]. No studies have indicated promotion of infection of IAV by defensins.

### 2.2. LL-37 and Influenza

A distinct group of antimicrobial peptides is called the cathelicidins and the one representative of this class in humans is LL-37. Recent reviews have discussed the extraordinary range of activities of LL-37, which include direct antimicrobial and antiviral activities, chemotactic activities for various immune cells, modulation of macrophage responses to inflammatory stimuli, and modulation of dendritic cell responses [26,27]. It is very likely that LL-37 participates in the host response to IAV. LL-37 resembles HNPs in that it is packaged in neutrophil granules and released upon cellular activation. LL-37 is also displayed on NETs where it can interact with IAV, which binds to NETs [6]. Like HBDs, LL-37 is produced by respiratory (and other) epithelial cells in response to various stimuli [26]. Leukotriene B4 (LTB4) has been shown to promote defense against IAV probably through its ability to stimulate release of LL-37 and β-defensins from respiratory epithelial cells [28].

As with other AMPs there is considerably more literature about the anti-bacterial activity than there is about the antiviral activity of LL-37. Recently, however, LL-37 has been found to inhibit several viruses including IAV, adenovirus, respiratory syncytial virus and HIV [29,30,31,32]. Several studies have recently established a role of LL-37 during IAV infection. Barlow et al. first demonstrated that LL-37 has direct antiviral activity against IAV and contributes to host defense against the virus in vivo both by limiting viral replication and virus-induced inflammation [33]. There is also a single murine cathelicidin, called CRAMP, which also inhibits IAV in vitro and in vivo [33,34]. Our laboratory then reported on the mechanism of antiviral activity of LL-37 that is distinct from that of collectins or defensins [34]. As with the defensins, direct incubation of the virus with LL-37 was needed to cause optimal inhibition of viral replication. Adding LL-37 to cells before or after infection was less effective. In contrast to the HNPs, LL-37 did not induce viral aggregation. In addition, LL-37 did not alter viral uptake by epithelial cells, although it markedly limited subsequent viral replication in the cells. Using electron microscopy, there was evidence of viral membrane degradation that could alter infectivity after viral internalization. This is consistent with the known membrane perturbing activities of LL-37. A similar finding was obtained by Currie et al. with respect to neutralization of RSV by LL-37 [35].

### 2.3. Other Peptides with Antiviral Activity for IAV

Fragments of other host defense molecules or peptides not commonly known to serve host defense functions have been showed to have AMP like activity with regard to bacteria and viruses.

#### 2.3.1. Histones

Histones are cationic peptides that resemble AMPs in some regards. There is increasing evidence that histones serve a host defense role vs. various organisms [36]. Histones are mainly known for their ability to bind to and regulate expression of DNA. However, histones are also found in cytosol and extracellular fluids where they appear to serve other functions, including antimicrobial activity. There are several reasons to believe histones may participate in the immune response during IAV infection. Like defensins and LL-37, histones are a major component of NETs [37]. In addition, histones are released into respiratory lining fluids during a variety of lung inflammatory states [38].

The bulk of studies of the antimicrobial activity of histones have focused on antibacterial activity. The lysine rich histones, H2A and H2B, have generally been found to have stronger anti-bacterial activity than the arginine rich histones H3 and H4 [39]. Several studies have demonstrated antiviral activity for histones as well [36,40]. We have recently reported that histones are able to neutralize influenza A viruses, with H3 and H4 having greater antiviral activity than H1, H2A and H2B [40]. The antiviral effect of the H4 was mediated by direct interaction with the virus rather than the host cells. In addition, H4 did not cause cell injury in a wide range of concentrations that reduced viral infectivity. H4 was able to strongly aggregate viral particles and this was associated with reduced uptake of the virus by target cells. Further studies of antiviral mechanisms of histones are warranted.

#### 2.3.2. Amyloid Beta (Aβ) Peptides

Aβ peptides are mainly known for their key role in the development of Alzheimer’s disease. However, Aβ peptides resemble some anti-microbial peptides or AMPs in their structure [41,42]. Aβ peptides are similar to the porcine AMP, protegrin, in ability to form channels in membrane structures, which is believed to be one of the anti-bacterial and anti-fungal mechanisms of AMPs. Soscia et al. demonstrated antibacterial and antifungal activity for Aβ peptides [43]. In addition, this study showed that Aβ isolated from the brain of AD patients had antimicrobial activity and that incubation of these brain derived samples with antibodies to Aβ ablated the antimicrobial activity. We recently reported that Aβ peptides also have antiviral activity against IAV [44]. A study by Bourgade et al. also showed inhibition of herpes simplex virus by Aβ peptides [45]. In our study and that of Soscia et al., Aβ1-42 was found to have greater antimicrobial or antiviral activity than Aβ1-40. We demonstrated that Aβ1-42, but not Aβ1-40, caused viral aggregation which appears to contribute to its antiviral effects. This implies a possible connection between the ability of Aβ1-42 to assemble into oligomers and its antiviral activity, since this peptide has a greater propensity to form oligomers and fibrils than Aβ1-40. An important recent study showed that Aβ peptides exert protective effects in mouse and worm models in vivo [46]. This study demonstrated that the antimicrobial mechanism of the peptides involved direct binding and then agglutination of the bacteria and fungi.

## 3. Immunomodulatory Effects of AMPs 

In addition to their direct antimicrobial and antiviral effects, AMPs have important modulatory effects on responses of many immune cells (see Table 1).

### 3.1. Effects on Viral Uptake, Respiratory Burst and NET Formation by Neutrophils

We have studied extensively the ability of AMPs to modulate viral interactions with neutrophils. HNPs, retrocyclins, histones and Aβ1-42 increase viral uptake by human neutrophils ([8,12,44] and unpublished data). We obtained similar results with human monocytes ([11,44], and unpublished data). It appears likely that the ability of these AMPs to increase viral uptake is linked to their viral aggregating activity. Consistent with this interpretation, LL-37 did not alter viral uptake by neutrophils [6]. The AMPs have varied effects on respiratory burst responses of neutrophils. IAV itself stimulates a respiratory burst response characterized by H_2_O_2_ production. Pre-incubation of IAV with HNPs reduced this H_2_O_2_ response, whereas histones, Aβ1-42, and LL-37 potentiated the response. LL-37 and Aβ1-42 also increased NET formation in response to IAV [47]. LL-37 has several known receptors on phagocytes, including formyl peptide receptor 2 (FPR2), CXCR2, the epidermal growth factor receptor and the P2X7 receptor [48]. By use of a specific inhibitor for FPR2 and other means we were able to show that the enhanced H_2_O_2_ and NET response to IAV caused by LL-37 is mediated by FPR2 [6].

### 3.2. Effects on Cytokine Responses to IAV

IAV is a potent stimulator of neutrophil IL-8 production and monocyte or macrophage pro-inflammatory cytokine production (e.g., TNF or IL-6). Pro-inflammatory cytokine production in vivo correlates with symptomatology and with lung injury caused by IAV. A major goal of current IAV research has been to reduce inflammatory effects of the virus to reduce lung injury [49]. In mouse models, lung injury can be reduced even when viral loads are unaffected in some cases [49]. LL-37 has been reported to reduce cytokine responses to various stimuli [50,51,52,53,54], and, indeed we found that it reduced neutrophil IL-8 responses to IAV and LPS [6]. We are in the process of studying the effects of the various AMPs on monocyte and macrophage cytokine responses to IAV. As noted, LL-37 treatment of mice infected with IAV reduces cytokine responses [33]. This effect appeared be in part independent of reduction of viral load by LL-37 in vivo.

HNPs released from dying neutrophils have been shown to mediate anti-inflammatory effects [55]. In addition, HNPs can be taken up by macrophages during bacterial infection where they inhibit macrophage inflammatory cytokine production by restricting protein translation [56]. The relevance of these findings to IAV infection has not been evaluated thus far. As noted also, deletion of HBD-1 resulted in greater inflammatory reaction to IAV, despite no change in viral titers. HBD 3 has been shown to have strong anti-inflammatory effects as well in an LPS model [57,58]. It will be of interest to test this peptide in the context of IAV infection. Even though Aβ1-42 has been extensively studied as a pro-inflammatory stimulus [59], we found that it reduced inflammatory cytokine production triggered by IAV in monocytes in vitro [44].

Overall, these results indicate that maximizing anti-inflammatory effects as well as antiviral activity should be considered when designing novel AMPs for treatment of IAV. A contrasting perspective has arisen from several studies in which increasing inflammation prior to IAV or SARS-CoV infection was protective [60,61,62]. In the study by Wohlford-Lenane et al. intranasal instillation of retrocyclin 1 alone induced lung inflammation and this was associated with protection vs. SARS-CoV. In another study, direct instillation of HNPs into the airway of mice had pro-inflammatory effects independent of infection [63]. Note that in other models listed above HNPs had anti-inflammatory effects. Hence, further study is needed to determine how to best administer AMPs in vivo and whether possible pro-inflammatory effects would be harmful or beneficial with respect to infection.

Although histones have antimicrobial and antiviral activities, they have been considered to instigate several systemic inflammatory diseases and tissue injuries, including sepsis, peritonitis, pancreatitis, stroke, acute lung injury, liver injury and kidney injury [36]. The injection of histones into mice can stimulate pro-inflammatory cytokine/chemokine release (e.g. IL-6, IL-8 and TNF) and leukocyte infiltration, resulting in sepsis-like pathology [64,65,66,67]. In these cases histones function as damage-associated molecular pattern molecules, and possible mechanisms for them to induce cytokine storm may be through the interactions with toll-like receptors (TLR), especially TLR2, -4 and -9, and the NLRP3 inflammasome [68]. Knowing the possible detrimental pro-inflammatory effects of histones, further study is needed to investigate the roles of histones during severe IAV and IAV-related lung injury.

### 3.3. Effects of AMPs on Adaptive Immune Responses

AMPs have been termed “alarmins”, in that they are able to trigger recruitment and activation of immune cells. AMPs modulate adaptive immune responses in various ways. This topic is beyond the scope of this paper; however, we refer the reader to other reviews [48,69]. As examples, HBDs and other defensins bind to receptors on lymphocytes and act as chemoattractants [70,71] and LL-37 facilitates presentation of antigens to by DCs to T cells [48,72]. α-defensins have been shown to potentiate neutralizing antibody responses to enteric viral infection [73]. The contribution of these activities to IAV infection have not been studied but certainly are valuable areas for future research.

### 3.4. Effects on Bacterial Superinfection

Bacterial superinfections are a major contributor to mortality during pandemics and seasonal epidemics of IAV [4]. These bacterial superinfections are difficult to treat with antibiotics. As a result there is great interest in identifying the cause of bacterial superinfection and how to intervene to prevent or treat these infections. The role of AMPs in bacterial superinfection, or for possible treatment of bacterial superinfection, has not been studied. However, given the combined antiviral and antibacterial activity of many AMPs they are attractive candidates for treatment of combined IAV and bacterial infection.

### 3.5. Interactions of AMPs with Other Host Defense Proteins

It is likely that the activity of AMPs in vivo reflects complex interactions among various AMPs and other host defense molecules [74,75]. We found that HNPs 1-3 bind to the lung host defense and surfactant regulatory protein, surfactant protein D (SP-D) [10,74]. The importance of this binding in vivo is not clear; however, HNPs inhibited antiviral activity of SP-D and caused precipitation of SP-D out of human BAL fluid. Since SP-D has anti-inflammatory properties in the uninfected and infected lung, this property of HNPs could account for inflammatory responses seen upon instillation of HNPs in the lung [63]. On the other hand, SP-D might aid in clearance of HNPs after neutrophil infiltration. Of note, we found that LL-37 and HBDs do not bind to SP-D [8], which may be advantageous when considering use of these peptides for therapy in the lung. Retrocyclins and LL-37 had additive antiviral effects when combined with SP-D [8,34].

Histones have been considered to augment inflammatory responses and promote thrombosis and thus can be responsible for IAV-related acute lung injury as noted above. There is evidence that other host defense proteins can bind histones and modulate their potential adverse effects. Little is known about the relationship between histones and SP-D. Evidence suggests that SP-D can simultaneously bind to both pathogens and NETs [64], and we recently found that SP-D can bind to histones and inhibit histone-induced respiratory burst in neutrophils (unpublished data). C-reactive protein (CRP), an acute phase reactant, is another host defense protein that is usually elevated during infections or inflammation, and CRP treatment in mice has been found to alleviate histone-induced toxicity, including endothelial damage, thrombosis and lung edema [76]. Other proteins like thrombomodulin have similar effects [77]. These findings suggest some possible modulators for histones, but further studies are needed [76].

## 4. Production of Modified Synthetic AMPs with Increased Antiviral Activity

### 4.1. Novel Cyclic or Alpha Defensins

There has been extensive research into ways to modify or mimic the structure of defensins in order to improve anti-bacterial or antiviral activity. Results of some of these studies are summarized in Table 2. Even single amino acid substitutions can potentiate antiviral activity. For instance, replacement of a single arginine with a lysine in retrocyclin 1 resulted in increased binding to, and inhibition of, HIV [14]. This modified retrocyclin also had increased IAV neutralizing activity [27]. Similarly, replacement of a single amino acid in HD5 resulted in increased activity vs. HSV [78]. The strong activity of retrocyclins also inspired construction of a panel of cyclic peptides by Ruchala and Lehrer [11]. The intent was to retain or improve on antiviral activities of naturally occurring retrocyclins with cyclic peptides that are easier to synthesize. These peptides were termed hapivirins and diprovirins and they incorporated systematic substitutions of key cationic or hydrophobic residues and also incorporated several synthetic amino acids. Several of the hapivirins and diprovirins had markedly increased neutralizing activity for IAV [11]. In addition, the peptides retained the properties of viral aggregation and increasing viral uptake by neutrophils. Like the retrocyclins, they did not interfere with the viral neutralizing activity of SP-D. In the case of the hapivirins, substitutions that increased hydrophobicity caused clear increase in antiviral activity, indicating that hydrophobicity is as important a feature as cationic charge for antiviral activity. The most active hapivirins and diprovirins also had strong ability to suppress TNF generation by human monocytes.

### 4.2. Novel β-Defensins

A recent paper by Zhao et al. took the approach of preparing a series of fragments of mouse beta defensin 4 and testing their activity against a panel of clinically important respiratory viruses, including human and avian IAV strains, SARS-CoV and MERS-CoV [79]. A 30 amino acid fragment of mBD4 (termed P9 by the authors) was found to have activity exceeding that of the full 40 amino acid protein both in vitro and in vivo in mice. In fact, 100% of mice survived lethal challenge with a mouse adapted H1N1 viral strain when they were pretreated with 50 µg/mouse of P9 intranasally. The P9 peptide also reduced lung inflammation and viral loads in the mice. P9 also increased survival of mice infected with a lethal dose of avian H5N1 or H7N9 and had in vitro antiviral activity against SARS-CoV and MERS-CoV. All of these respiratory viral strains are of great concern due to pandemic potential and severe outcomes of infection in humans. The mechanism of action of P9 was found to include binding to H1N1 IAV, entering the cell along with the virus and impairing endosomal acidification and hence viral escape from endosomes. This study provides and interesting example of a shortened fragment of a naturally occurring defensin having greater activity than the parent peptide.

### 4.3. LL-37 Derived Peptides

In a similar vein, shortened or subtly modified version of LL-37 have been tested for antiviral and antibacterial activity. A 20 amino acid fragment, termed GI-20, was found to have retained the antiviral activity of the full molecule against seasonal IAV [80] and against HIV [81]. Another fragment called K-13 had no activity for IAV, despite the fact that it inhibits HIV and has anti-bacterial activity as well [82,83]. Two N-terminal fragments (LL-23 and LL-23V9) that have anti-bacterial activity [84] failed to inhibit IAV [85]. The GI-20 fragment incorporates the central helix of LL-37 and differs by one amino acid from the LL-37 sequence (isoleucine 13 is changed to glycine). Of note, pandemic H1N1 of 2009 was inhibited in vitro by GI-20, but not by LL-37 or the other peptides [85]. This provides another example of a shortened version of a natural AMP with increased antiviral activity compared to the parent peptide. GI-20 also retained the ability of LL-37 to increase neutrophil respiratory burst and NET responses to IAV, while also suppressing neutrophil IL-8 response to the virus [80]. The ability of GI-20 to increase virus induced respiratory burst and NET responses was also mediated through binding to FPR2. Using molecular modeling GI-20 was found to incorporate the domain of the protein predicted to bind to the FPR2 receptor.

### 4.4. Lactoferrin and Bacterial/Permeability Increasing Protein (BPI) Based Peptides

Lactoferrin is an evolutionarily conserved innate immune protein present in breast milk and other mucosal secretions that is cationic and has activity against bacteria and several viruses, including IAV [86]. Lactoferrin has two lobes (the N and C lobes) both of which contain an iron binding site. Although the N and C lobes have similar structures they have distinct sequences within them. The two lobes can be separated by proteolytic cleavage and shown to have distinct functional activities [87]. A recent study by Ammendolia et al. showed that all of the anti-influenza activity of bovine lactoferrin is in the C lobe and that shorter peptide domains within the C lobe have picomolar activity against IAV [88]. These peptides had greater antiviral activity in vitro and greater selectivity indices (a measure that combines antiviral activity and cellular cytotoxicity) than the whole lactoferrin protein. The antiviral activity of lactoferrin was related to binding to the HA_2_ subunit of the viral hemagglutinin (which contains the fusion domain).

A 27 amino acid N-terminal fragment of human BPI was recently shown to inhibit various IAV strains (including H1N1, H3N2 and H5N1 strains) in vitro through a direct action on the virus [89]. Of interest, the murine homolog of this peptide lacked anti-IAV activity. In addition, the human peptide inhibited IL-6 and TNF responses of monocytes triggered by IAV.

## 5. Potential for Resistance of IAV to AMPs

Mechanisms of resistance to AMPs have been well described in bacteria; however, there are no similar data for viruses. As noted above we found that pandemic IAV was not inhibited by LL-37 (although the GI-20 fragment was inhibitory). Similarly we found that the murine cathelicidin CRAMP [85] and human histone H4 did not inhibit pandemic H1N1 of 2009 [40]. HNP-1 had reduced activity for the pandemic strain as well. The mechanism of resistance of the pandemic strain is not clear since all of these AMP have activity against seasonal or mouse adapted strains of IAV. Using reverse genetics a viral strain was prepared having only the HA of the pandemic strain but all other gene segments from a seasonal H1N1 strain and this re-assorted strain was sensitive to LL-37, CRAMP and histone H4. This indicates that resistance is not mediated by the hemagglutinin of the pandemic strain. Further studies will be important to determine which components of the pandemic strain confer resistance to several AMPs.

## 6. Induction of Endogenous AMPs

One of the challenging features of developing AMPs as therapeutics is determining how to administer them. As noted above, there is evidence that direct administration into the airway may induce inflammatory responses. Another approach under exploration is increasing endogenous production of AMPs through various means [90]. Results of these studies are summarized in Table 3. As noted, LTB-4 can potentiate generation of both LL-37 and HBD in the respiratory tract [28,91]. LL-37 generation by epithelial cells is regulated by vitamin D; hence, repletion of vitamin D may have host defense benefits. LL-37 generation is also enhanced by histone deacetylase inhibitors (e.g. phenylbutyrate) [92]. This approach was found to improve outcomes when added to standard antibiotic treatment of mycobacterium tuberculosis infection in humans [93]. An interesting report also showed that supplementation with the amino acid isoleucine can increase HBD expression [94]. Further studies of this phenomenon will be of special interest given the low toxicity of this approach. IL-17 and IL-22 have been shown to induce expression of HBD and S100 peptides (another group of AMPs) in human keratinocytes [95]. One paper reported on endogenous generation of retrocyclins in human cells after exposure to aminoglycoside antibiotics [96]. Human cells actually contain the gene for retrocyclins but they are not expressed due to a premature stop codon in the signal sequence [97]. Clearly the study of AMP induction is in its infancy. However, there are several tantalizing findings that certainly support further exploration of this approach.

## 7. Conclusions

IAV causes many deaths or severe illness every year in seasonal outbreaks and has the potential to cause massive morbidity and mortality during pandemics. Resistance to two classes of drugs that are currently used for IAV treatment has been emerging, so there has been extensive interest in developing new antiviral treatment for this virus. Deaths caused by IAV infection mostly resulted from acute lung injury, systemic inflammation or bacterial superinfection, suggesting that new treatments with anti-viral, anti-bacterial and anti-inflammation effects would be ideal. AMPs are antimicrobial peptides that not only play important roles as host defense against pathogens but also modulate inflammatory responses, and thus they are potential candidates for IAV treatment. We discussed the interactions of two classes of classical AMPs (defensins and cathelicidins) and two non-classical AMPs (histones and Aβ) with IAVs in this paper. For the most part, these AMPs possess anti-IAV activity by direct interacting with the virus, although in some instances direct interactions with mammalian cells may contribute to antiviral effects. All of AMPs we discussed also have immunomodulatory effects, with some up-regulating and others down-regulating inflammation. We provide some examples (e.g., hapivirins and diprovirins or GI-20) in which novel synthetic AMPs or AMP fragments have improved anti-IAV or immunomodulatory activities, suggesting that modification of AMPs is an attractive strategy. Further studies are required to determine the interactions between AMPs and other host defense proteins in the lung, the best methods to administer or induce generation of AMPs, and to explain instances of resistance of pandemic IAV strains to AMPs.

## Figures and Tables

**Table 1:** Antiviral and immunomodulatory activity of AMPs with respect to IAV.

AMP	Principle Human Lung Source	Antiviral Activity ^a^	Immune Modulation ^b^
α Defensin	Neutrophil	Seasonal strains: 3+ Pandemic strains: 1+	*Neutrophils*: increased viral uptake, reduced H_2_O_2_ response *Monocytes*: cytokines reduced
β Defensin	Epithelial Cells	Seasonal strains: 2+ Pandemic strains: ND	*Neutrophils*: increased viral uptake *Monocytes*: cytokines reduced
θ Defensin	Not present in humans	Seasonal strains: 4+ Pandemic strains: ND	*Neutrophils*: increased viral uptake *Monocytes*: cytokines reduced
LL-37	Neutrophils, macrophages, epithelial cells	Seasonal strains: 3+ Pandemic strains: +/−	*Neutrophils*: increased H_2_O_2_ and NETs, reduced IL-8 *Monocytes*: cytokines reduced
Amyloid Beta (Aβ)	Unknown	Seasonal strains: 2+ Pandemic strains: 2+	*Neutrophils*: increased viral uptake, increased H_2_O_2_ and NET response *Monocytes*: cytokines reduced
Histones H3 and H4	NETs and necrotic cells	Seasonal strains: 3+ Pandemic strains: +/−	*Neutrophils*: increased viral uptake, increased H_2_O_2_ response
Lactoferrin peptides	Neutrophils	Seasonal strains: 4+ Pandemic strains: ND	ND

^a^ Antiviral activity for seasonal and pandemic IAV strains are indicated by a scale of 0 to 4+ to give a general idea of the relative potency of the different peptides. In some instances activity against pandemic strains were not determined (ND). ^b^ Results for immune modulation are not comprehensive.

**Table 2:** Therapeutic Directions: Creation of Novel AMPs.

Prototype Peptide	Antiviral Activity	Immune modulation
β Defensin	Novel p9 peptide has increased antiviral activity against human and avian IAV strains	Mediate anti-inflammatory effects
Cyclic defensins	Hapivirins and Diprovirins have increased antiviral activity against seasonal IAV	Cause increased neutrophil uptake compared with HNPs and suppress TNF generation by monocytes
LL-37	GI-20 gains activity against pandemic strain	Have similar immunomodulatory effects to LL-37
Lactoferrin	Shorter peptide fragments show increased anti-IAV activity for seasonal and mouse adapted strains	Not tested
BPI	27 amino acid N-terminal fragment of human BPI inhibits infectivity of various IAV strains	Inhibit monocyte cytokine production in response to IAV

**Table 3:** Therapeutic Directions: Increase endogenous AMP generation.

Mediator	AMPs Effected
LTB4	Increase LL-37 and β Defensin generation
HDAC inhibitors	Increase LL-37 generation
Vitamin D	Increase LL-37 generation
Isoleucine	Increase HBD expression
IL-22, IL-17	Increase AMP expression
